# Haitian Variant *Vibrio cholerae* O1 Strains Manifest Higher Virulence in Animal Models

**DOI:** 10.3389/fmicb.2019.00111

**Published:** 2019-02-05

**Authors:** Priyanka Ghosh, Ritam Sinha, Prosenjit Samanta, Dhira Rani Saha, Hemanta Koley, Shanta Dutta, Keinosuke Okamoto, Amit Ghosh, T. Ramamurthy, Asish K. Mukhopadhyay

**Affiliations:** ^1^Division of Bacteriology, National Institute of Cholera and Enteric Diseases, Kolkata, India; ^2^Collaborative Research Center of Okayama University for Infectious Diseases in India, Kolkata, India; ^3^Center for Human Microbial Ecology, Translational Health Science and Technology Institute, Faridabad, India

**Keywords:** cholera, *V. cholerae*, El Tor, Haitian variant, pathogenesis

## Abstract

*Vibrio cholerae* causes fatal diarrheal disease cholera in humans due to consumption of contaminated water and food. To instigate the disease, the bacterium must evade the host intestinal innate immune system; penetrate the mucus layer of the small intestine, adhere and multiply on the surface of microvilli and produce toxin(s) through the action of virulence associated genes. *V. cholerae* O1 that has caused a major cholera outbreak in Haiti contained several unique genetic signatures. These novel traits are used to differentiate them from the canonical El Tor strains. Several studies reported the spread of these Haitian variant strains in different parts of the world including Asia and Africa, but there is a paucity of information on the clinical consequence of these genetic changes. To understand the impact of these changes, we undertook a study involving mice and rabbit models to evaluate the pathogenesis. The colonization ability of Haitian variant strain in comparison to canonical El Tor strain was found to be significantly more in both suckling mice and rabbit model. Adult mice also displayed the same results. Besides that, infection patterns of Haitian variant strains showed a completely different picture. Increased mucosal damaging, colonization, and inflammatory changes were observed through hematoxylin-eosin staining and transmission electron microscopy. Fluid accumulation ability was also significantly higher in rabbit model. Our study indicated that these virulence features of the Haitian variant strain may have some association with the severe clinical outcome of the cholera patients in different parts of the world.

## Introduction

Disease cholera continues to be a severe public health threat in the developing countries. Regardless of the availability of prevention, control and treatment management, the global burden of cholera is still alarming. An estimated annual cholera cases varies between 1.3 and 4.0 million in 69 cholera-endemic countries, with 21,000 to 143,000 deaths in a year (Ali et al., 2015). WHO reported 1,227,391 cases and 5654 deaths in 34 countries (World Health Organization [WHO], 2018c). Several devastating cholera outbreaks have been reported worldwide in the recent past. Among them, the Haitian cholera outbreak in the year 2010 placed this ancient disease once again at the front line of the global public health agenda (Piarroux et al., 2011; World Health Organization [WHO], 2011, 2012). During the year 2016–2017 more than 20,000 suspected cholera cases with over 400 deaths were reported from South Sudan (Weekly bulletin on outbreaks and other emergencies, World Health Organization [WHO], 2018b). During January 2017, more than 1,000 deaths were reported from Somalia and in the Democratic Republic of the Congo (Cholera outbreak updates, World Health Organization [WHO], 2018a, Weekly bulletin on outbreaks and other emergencies, World Health Organization [WHO], 2018b). Currently, war-torn Yemen is encountering the world’s largest cholera outbreak. Since April 2017, more than 1 million suspected cases and over 2,000 deaths have been reported (Camacho et al., 2018).

There are more than 200 serogroups of *V. cholerae* and among them only O1 and O139 serogroups can cause epidemic and pandemic cholera (Kaper et al., 1995; Safa et al., 2010). The serogroup O1, based on distinct phenotypic and genetic characteristics, can be divided into classical and El Tor biotypes (Banerjee et al., 2014). Till date, the world has experienced seven pandemics of cholera. Among them, the first six pandemics were caused by classical biotype whereas the ongoing seventh pandemic is due to the El Tor biotype (Safa et al., 2010; Zhang et al., 2014; Hu et al., 2016; Weill et al., 2017). The seventh cholera pandemic is remarkably different from the others in terms of its rapid global spread and long duration of more than 50 years (Mutreja et al., 2011). The seventh and ongoing cholera pandemic also has spawned several novel El Tor variants of *V. cholerae* O1 that have made their appearance in many Asian and African countries (Safa et al., 2008; Clemens et al., 2017). These include, the Matlab variant, Mozambique variant, the altered El Tor variant and the recently Haitian variant (Nair et al., 2002, 2006; Faruque et al., 2007; Chin et al., 2011). Among these, Haitian variant has turned out to be most deadly affecting around 0.8 million people along with around 9000 deaths in Haiti (Piarroux et al., 2011). Whole genome sequencing analysis of *V. cholerae* isolates from Haiti revealed several unique genetic changes (Chin et al., 2011). These include structural variation in superintegron, VSP-2, and SXT as well as single nucleotide polymorphisms (SNPs) in the *ctxB, tcpA, rtxA*, and *rstB2* regions, which are the key factors for *V. cholerae* colonization and pathogenesis (Son et al., 2011; Talkington et al., 2011). Remarkably, the Haitian strains with its newer genetic makeup are different from those exists in the nearby countries, i.e., Latin America and U.S. Gulf coast but had close similarity with South Asian isolates (Chin et al., 2011; Hendriksen et al., 2011; Reimer et al., 2011). In recent years, Haitian variant strains have been isolated in different parts of Asia and Africa.

Based on the retrospective genetic analysis of *V. cholerae* O1 strains from Kolkata and Delhi, it was shown that Haitian variant emerged from the Indian subcontinent much before the Haitian outbreak in 2010 (Naha et al., 2012; Ghosh et al., 2014a,b, 2017), and subsequently spread to different parts of India (Samanta et al., 2018), Nigeria (Marin et al., 2013), Mexico and Zambia (Marin and Vicente, 2012; Díaz-Quiñonez, 2017). Although there are several reports regarding the spread of the *V. cholerae* strains with Haitian genetic traits in different parts of the world but there is a dearth of information on clinical consequence of such genetic changes as the clinical relevance of these genetic changes in Haitian variant has not been studied in detail. To investigate the importance of such genetic modifications in Haitian variant strain, we performed *in vivo* colonization and virulence assays using mouse and rabbit models and compared its pathogenic abilities with canonical El Tor.

## Materials and Methods

### Reagents

All chemicals were of analytical grade and obtained from Sigma-Aldrich Chemicals (St. Louis, MO, United States). Thiosulfate-Citrate Bile salt Sucrose agar (TCBS) was from Eiken (Japan) and Luria Bertani (LB) medium was from Difco (United States).

### Bacterial Strains, Media, and Growth Condition

Representative *Vibrio cholerae* O1 strains El Tor (V100 and V114; isolated during 1990) and Haitian variants (IDH00990 and IDH02003; isolated during 2008) from the strain repository of ‘National Institute of Cholera and Enteric Diseases’ (NICED), Kolkata, India, were used in this study. These strains were isolated from the cholera patients admitted to the Infectious Diseases and Beliaghata General (ID&BG) Hospital, Kolkata, India. Detailed phenotypic and genetic characteristics of these strains are presented in Table 1. *V. cholerae* strains were grown in Luria-Bertani (LB) medium containing 1% NaCl at 37°C with shaking. Strains were stored at −80°C in LB containing 20% glycerol (V/V).

**Table 1:** Description of strains used in this study.

Characteristics	V100 O1 Ogawa (1990)	V114 O1 Ogawa (1990)	IDH0990 O1 Ogawa (2008)	IDH02003 O1 Ogawa (2009)
***Phenotypic assays***				
Polymyxin B susceptibility assay	R	R	R	R
Voges Proskauer test	+	+	+	+
***Genetic tests (PCR)***				
*ctxB*	EL	EL	HT	HT
*tcpA*	EL	EL	HT	HT
*rtxA*	EL	EL	HT	HT
*ctxA*	+	+	+	+
*Cep*	+	+	+	+
*Zot*	+	+	+	+

*R, resistant; CL, classical; HT, Haitian; EL, El Tor.*

### Growth Curve Analysis

Medial growth curve analysis was performed with canonical El Tor and Haitian variant strains. Single colony was inoculated in 5 mL of LB broth and grown overnight at 37°C under shaking condition. Overnight cultures were sub-cultured in fresh 25 mL LB broth in a sterile glass conical flask at a dilution of 10^3^. The cultures were further incubated at 37°C and the O.D_600_ was measured at every 1 h time interval up to 14 h (Medrano et al., 1999; Chatterjee et al., 2017).

### Animals

New Zealand white rabbits of either sex weighing 2.0–2.5 kg, 4 to 5 days-old suckling BALB/c mice and 4–5 weeks adult BALB/c mice were used in this study (Table 2). All animals were maintained under specific pathogen free condition in ventilated cages in the Animal House facility of NICED and provided with sterilized food and water.

**Table 2:** List of animals used in this study.

Animal model	Age/weight/size/ gender	Purpose	Groups	
BALB/c suckling mice	4–5 days old Any gender	Colonization	Groups 1–5 (PBS, V100, V114, IDH00990, IDH02003)	*n* = 9 in each group
New Zealand white Rabbit	2–2.5 kg Any gender	Enterotoxicity (ileal loop, colonization, histopathology)	*Groups 1–5* *(PBS, V100, V114, IDH00990, IDH02003)*	*n* = 3
BALB/c adult mouse	4–5 week, 18–20 gm Any gender	Disease persistency, colonization, and histopathology	Groups 1–5 (PBS, V100, V114, IDH00990, IDH02003)	*n* = 21 in each group (7 days shedding and colonization)

### Ethics Statement for the Animal Experimentation

Animal experiments were conducted following the standard operating procedure framed by Committee for the purpose of Control and Supervision of Experiments on Animal (CPCSEA) (Approval No. PRO/111/Nov2014-Dec2016), Ministry of environment and forest, Government of India. The animal experimental protocol was approved by the Institutional Animal Ethics Committee of NICED.

### Bacterial Cultures for Animal Experiments

Overnight cultures of *V. cholerae* strains were diluted 1:1000 in fresh LB medium supplemented with 100 μg of streptomycin/ml (LBS) and grown at 37°C with shaking to mid-log phase. Bacterial cultures were centrifuged, the pellets washed twice with ice cold phosphate-buffered saline (PBS), and then suspended in cold PBS (1 × 10^9^ CFU/50 μl).

### Infant Mouse Colonization Assay

Colonization assay was performed in suckling mouse model with the modified protocol described previously (Camilli and Mekalanos, 1995; Angelichio et al., 1999). Briefly, 4 to 5 day-old suckling BALB/c mice were separated from their mothers 1 h prior to inoculation with *V. cholerae*. Each mouse was then intragastrically inoculated with 50 μl of the diluted bacterial culture. Negative control mice were fed with 50 μl of PBS only. The bacterial titer in each inoculum was determined by plating serial dilutions on LBS agar plates. Infected mice were kept at 26°C separated from their mother. These were sacrificed 18 h after infection by cervical dislocations under isoflurane. Their intestines were then removed and mechanically homogenized in cold PBS (100 μl) using a tissue homogenizer at 4°C (POLYTRON PT1600E, Kinematica AG). Serial dilutions of the homogenates were then plated onto LBS agar to enumerate viable *V. cholerae* cells and expressed in log of Colony Forming Unit/gram (CFU/gram) of the intestine. Values were calculated as mean ± SD. Nine mice per group were used in this study.

### Rabbit Ileal Loop Assay for Detection of Enterotoxigenecity

The rabbit ileal loop test was performed as described previously (Koley et al., 1999). New Zealand white rabbits were fasted for 48 h before the surgery and only fed water *ad libitum*. Rabbits were anesthetised by intramuscular injection of ketamine (35 mg/kg body weight) and xylazine (5 mg/kg body weight). A laparotomy was performed, and the ileum was washed and ligated into discrete loops of about 10 cm. Each segment was separated by uninoculated segments of 1 to 2 cm. Test loops were inoculated with 10^9^ CFU of the challenge strain (V100, V114, IDH00990, IDH02003) in PBS. Negative-control loops were infused with PBS. The intestine was put back into the peritoneal cavity, animals were sutured and returned to their cages. After about 18 h, the animals were sacrificed with sodium pentobarbital (150 mg/kg) and the abdomens were re-opened. Loops were taken out. The volume of the accumulated fluid and the length of the loops were measured. The extent of the fluid accumulation (FA) was expressed as loop fluid volume (ml)/length (cm) ratio. Values were expressed as mean ± SD (three rabbits per group). Using the same model the bacterial colonization and histopathology were also performed. Bacterial colonization was quantified as mentioned earlier.

### Streptomycin Treated Adult Mouse Model

Pathogen free, 4 to 5-week-old BALB/c mice (five groups consisting three mice/group) were orally treated with 500 μl of sterile water containing 20 mg of streptomycin for 7 days as described previously (Sinha et al., 2016). Water and food were withdrawn 4 h before the streptomycin treatment as well as before infecting the rabbits with *V. cholerae*. The mice were then infected orally with 50 μl of 8.5% (wt/vol) sodium bicarbonate immediately followed by 50 μl of PBS containing 10^9^ CFU test strains of *V. cholerae* (V100, V114, IDH00990, and IDH02003). Negative control mice were fed with 50 μl of PBS only. Food was then immediately offered to all animals. To analyze bacterial shedding pattern, stool samples from three mice from each group were collected at a particular time up to 7 days.

To analyze intestinal colonization a similar but separate experiments like above (five groups; *n* = 21) were performed. Mice were sacrificed by cervical dislocations under anesthesia (isoflurane inhalation). The small intestines were removed aseptically from three animals of each group and homogenized in 4°C cold PBS. This procedure was repeated for 7 days. After dilution, homogenized stool samples and intestinal soups were diluted and plated on LBS agar. *V. cholerae* was enumerated in log of Colony Forming Unit/gram (CFU/gm) of stool and intestine. Values were expressed as mean ± SD. Intestinal tissues of these infected mice were used also for hematoxilin and eosin (H/E) staining and transmission electron microscopic analysis.

### Histopathology and Transmission Electron Microscopy (TEM)

Histopathology was performed with the intestinal tissues of *V. cholerae* infected and uninfected adult mice and also of the rabbit models that were used for colonization assays. Briefly, tissue sections were embedded in paraffin block and then, 2–3 μm thick sections from paraffin-embedded intestinal segments were cut, placed on glass slides, and stained with H/E staining. All samples were examined by light microscopy (Leica DMLB), and digital photographs of stained tissues were taken at a magnification of 40x. H/E strained intestinal samples were then analyzed through Leica Quin Digital Image processing software and also by a pathologist blinded to the experimental sample set (D.R. Saha, National Institute of Cholera and Enteric Diseases, India). Scoring of the histological sections was also done (Barthel et al., 2003).

For TEM, small pieces of tissues were fixed in 3% cacodylate buffered glutaraldehyde (pH 7.4) for 4 h which were then rinsed in 0.1 M cacodylate buffer overnight. Post-fix specimens were incubated for 1 h in cacodylate buffer containing 1% Osmium tetroxide and then rinsed twice in 0.1 M cacodylate buffer (10 min each). Tissue samples were dehydrated with the gradient alcohol concentration. After that, infiltration with resin and embedding were done using BEEM capsule following standardized laboratory method. Tissue blocks were prepared and images were taken at 100 kV (FEI, Tecnai 126 12, BIOTWIN, Netherlands). TEM analysis was also performed by a pathologist who was blinded to the experimental protocol.

### Statistical Analysis

Data were expressed as mean ± standard deviation (SD). Statistical analysis was evaluated by Kruskal–Wallis one way ANOVA or two way ANOVA using GraphPad Prism software, version 5 (GraphPad Software, Inc., San Diego, CA, United States); ^∗^*p* < 0.05 and ^∗∗^*p* < 0.005 were considered as statistically significant.

## Results

### Growth Pattern of El Tor and Haitian Variant

**FIGURE 1:**
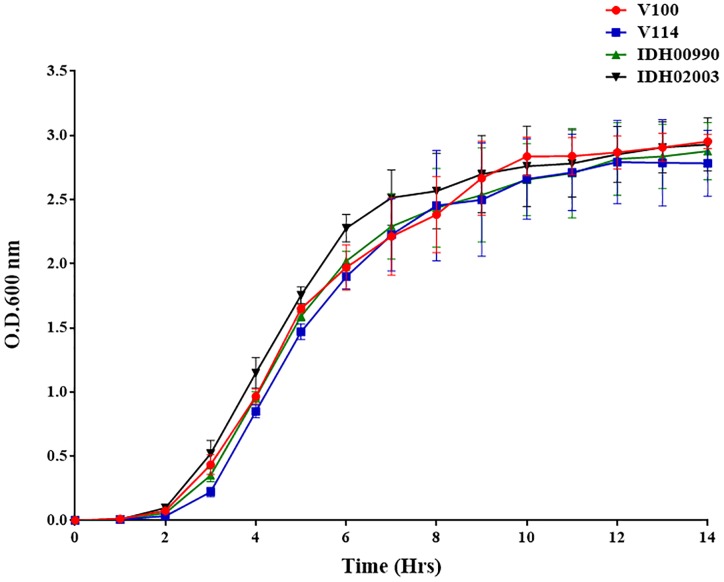
*Vibrio cholerae growth* curve of El Tor strains (V100, V114) and Haitian variant Strains (IDH00990 and IDH02003) were grown in Luria-Bertani broth for 14 h, and the optical density was measured every hour at 600 nm.

The growth kinetics of both El Tor representatives (V100, V114) and Haitian variants (IDH00990 and IDH02003) were found to be predominantly sigmoidal. This result indicates the consistency in their growth patterns. Although the *V. cholerae* strains had different genetic backgrounds, their growth pattern did not differ significantly (Figure 1).

### Intestinal Colonization of El Tor and Haitian Variant Strains in Suckling Mouse Model

**FIGURE 2:**
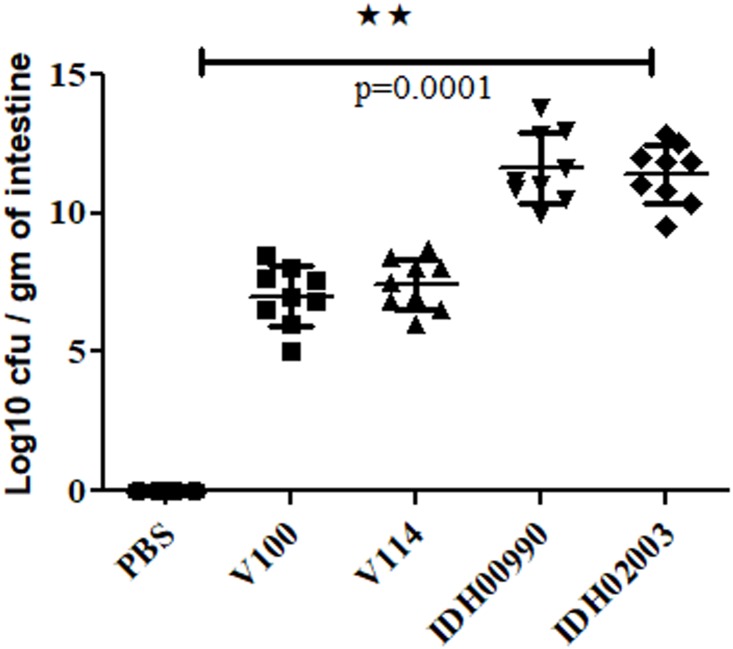
Assay to measure the colonization potential in infant mice model. Log phase *V. cholerae* El Tor strains V100 (1 × 10^9^), V114 (1 × 10^9^) and Haitian variant strains IDH00990 (1 × 10^9^), IDH02003 (1 × 10^9^) were inoculated and CFU/gm of intestine were measured as described in Section “Materials and Methods.” Data are expressed as mean ± SD of nine mice in each group. In all cases Haitian variant strains displayed significant higher colonization in comparison with El Tor strains. ^∗∗^highly significant.

Intestinal colonization in suckling mouse was examined to find the colonization efficiency of El Tor and Haitian variant strains. In suckling mouse model, we found that Haitian variants (IDH00990 and IDH02003) had significantly higher colonization than the canonical El Tor strains (V100 and V114) after 18 h of infection. Mean Log_10_CFU/gm of intestine count of V100 (7 ± 1) and V114 (7.4 ± 0.9) yielded significantly lower colonization efficacy compared to IDH00990 (11.64 ± 1.27) and IDH02003 (11.42 ± 1, *p* = 0.0001) (Figure 2).

### Enterotoxicity of El Tor and Haitian Variants in a Rabbit Model

**FIGURE 3:**
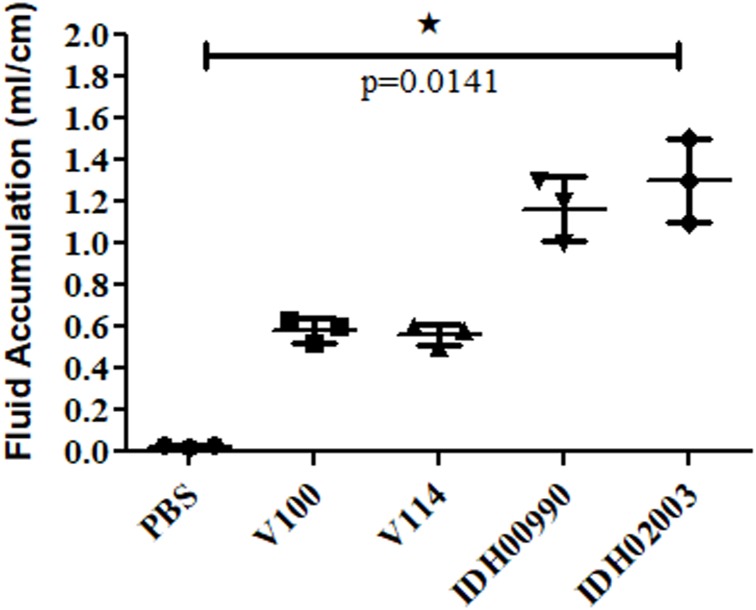
Graphical representation of comparative fluid accumulation ratio of El Tor strains V100, V114 and Haitian variant strains IDH00990, IDH02003 in rabbit model. Data are expressed as mean ± SD of three different rabbit in each group. Significantly higher fluid accumulation was observed for Haitian variant strains IDH00990 and IDH02003. ^∗^significant.

**FIGURE 4:**
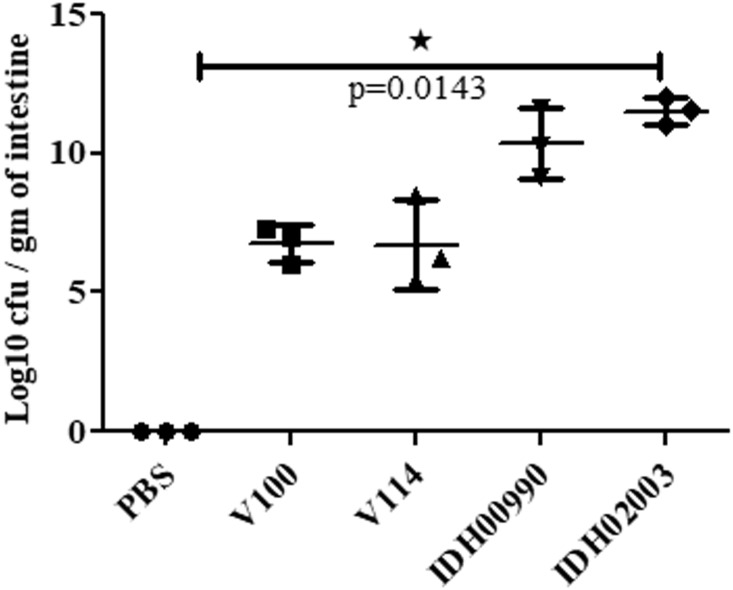
Assay optimization for measure of colonization potential in rabbit model. Log phase *V. cholerae* El Tor strains V100 (1 × 10^9^), V114 (1 × 10^9^) and Haitian variant strains IDH00990 (1 × 10^9^), IDH02003 (1 × 10^9^) were inoculated and CFU/gm of intestine were measured as described in Section “Materials and Methods.” Data are expressed as mean ± SD of three different rabbit from each group. Significantly higher colonization was observed for IDH00990 and IDH02003 strains. ^∗^significant.

**FIGURE 5:**
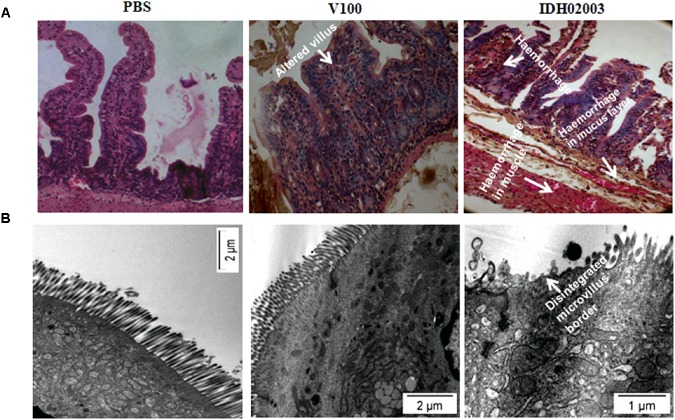
**(A)** Hematoxylin and Eosin stained sections of rabbit intestine challenged with *V. cholerae* El Tor strain V100 (1 × 10^9^) and Haitian variant IDH02003. Although widely dilated villi with congestion and scattered hemorrhage was seen with V100 but as can be seen the hemorrhagic change in mucosa and sub mucosa with structural alteration and disruption of villous surface was more with IDH02003 in the same time period. **(B)** Transmission electron microscopy of rabbit intestine challenged with *V. cholerae* El Tor strain V100 (1 × 10^9^) and newer variant IDH02003 respectively. TEM study showed damaged microvillus structure with IDH02003 but no such change was seen with V100 treated section.

Less fluid accumulation (FA) was observed with El Tor strains compared to the Haitian variant. This test depicted FA ratio of 0.58 ± 0.06 and 0.56 ± 0.05 with strains V100 and V114, respectively, while the strains IDH00990 and IDH02003 displayed a significantly higher FA ratio of 1.16 ± 0.15 and 1.3 ± 0.2 (*p* = 0.0141), respectively (Figure 3). In the colonization assay, Log_10_CFU/gm of intestine count in V100 (6.7 ± 0.68) and V114 (6.72 ± 1.59) showed significant decrease in the colonization ability as compared to and IDH00990 (10.37 ± 1.25) and IDH02003 (11.51 ± 0.5, *p* = 0.0143), respectively (Figure 4). Intestinal tissues from the same rabbits were used for H/E analysis and TEM. Widely dilated villi were observed in the H/E stained rabbit intestine infected with V100. Congestion and scattered hemorrhage were also found. On the other hand, hemorrhagic changes in both mucous and sub-mucous layer were seen in the case of IDH02003 infected tissue. Structural alteration and disruption of the villous surface was clear (Figure 5A). TEM analysis showed the damaged microvillus structure with IDH02003 but no such changes were seen with V100 treated sections (Figure 5B).

### Pattern of Bacterial Shedding and Intestinal Colonization of El Tor and Haitian Strains in Adult Mouse Model

**FIGURE 6:**
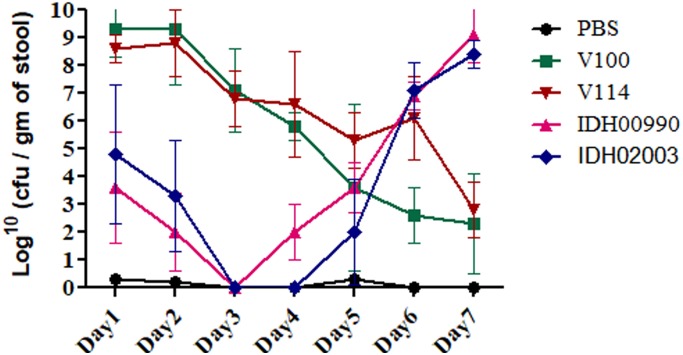
Shedding of *V. cholerae* strains in stools of adult mice challenged with El Tor and Haitian variant strains. Log phase *V. cholerae* El Tor strains V100 (1 × 10^9^), V114 (1 × 10^9^) and Haitian variant strains IDH00990 (1 × 10^9^), IDH02003 (1 × 10^9^) were inoculated and CFU/gm of stool was measured for each day over a 7-day period as described in Section “Materials and Methods.” Data are expressed as mean ± SD of three different mice from each group. Bacterial shedding prevailed throughout the 7-day observation period. V100 displayed peak shedding at day 1 followed by a gradual decline. In contrast, bacterial shedding of IDH02003 totally halted on days 3 and 4, followed by enhanced shedding up to day 6. V114 and IDH00990 followed almost similar pattern of V100 and IDH02003 respectively.

Altered genomic features, higher colonization ability and FA effects of Haitian variant strains driven us to compare the disease persistency of El Tor and Haitian variants. Bacterial shedding prevailed throughout the 7 days. V100 displayed a peak fecal shedding on day 1 with ≈9.3 log_10_CFU/gm of stool. This was followed by a gradual declining trend. No bacterial shedding of IDH02003 was observed on days 3 and 4. Bacterial shedding was again observed from day 5 with a rise on day 6. This shedding profile was almost identical with the other strain IDH00990. The shedding profiles of V100 and V114 showed a gradual declining phase (Figure 6). Our results depicted persistent diarrhea by Haitian variants which resembles the infection pattern of non-O1 and non-O139.

**FIGURE 7:**
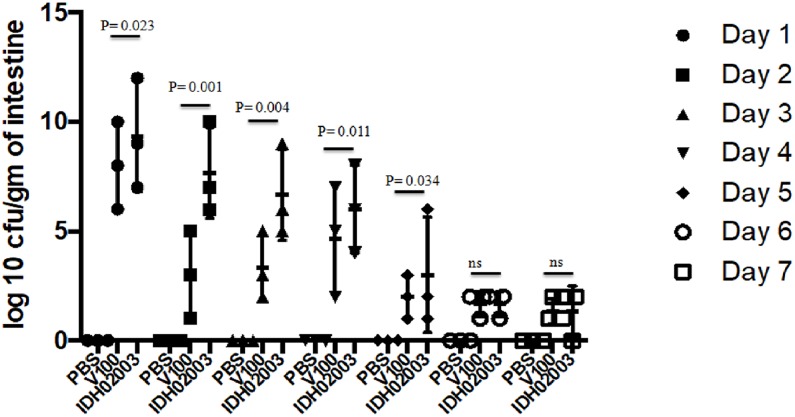
Assay to study the 7-day colonization potential in adult mouse model. Log phase *V. cholerae* El Tor strain V100 (1 × 10^9^) and Haitian variant strain IDH02003 (1 × 10^9^) were inoculated and CFU/gm of intestine was measured for each day in the 7-day period as described in Section “Materials and Methods.” Data are expressed as mean ± SD of three different mice from each group. In both cases colonization decreased over the 7-day period manner but for El Tor strains a rapid decrease was seen after day 2.

Though bacterial shedding was observed for 7-days, but there was no shedding on days 3 and 4 in Haitian variant infected mice. To confirm this persistent diarrheal pattern, we performed a 7-day colonization assay. Colonization also occurred throughout the 7 days in both El Tor and Haitian variant strains. Log_10_CFU/gm of intestine count in IDH02003 was significantly higher up to day 5 in comparison to V100 (Figure 7).

### Examination of the Tissue Damaging Capacity of the El Tor and Haitian Variant Strains

**FIGURE 8:**
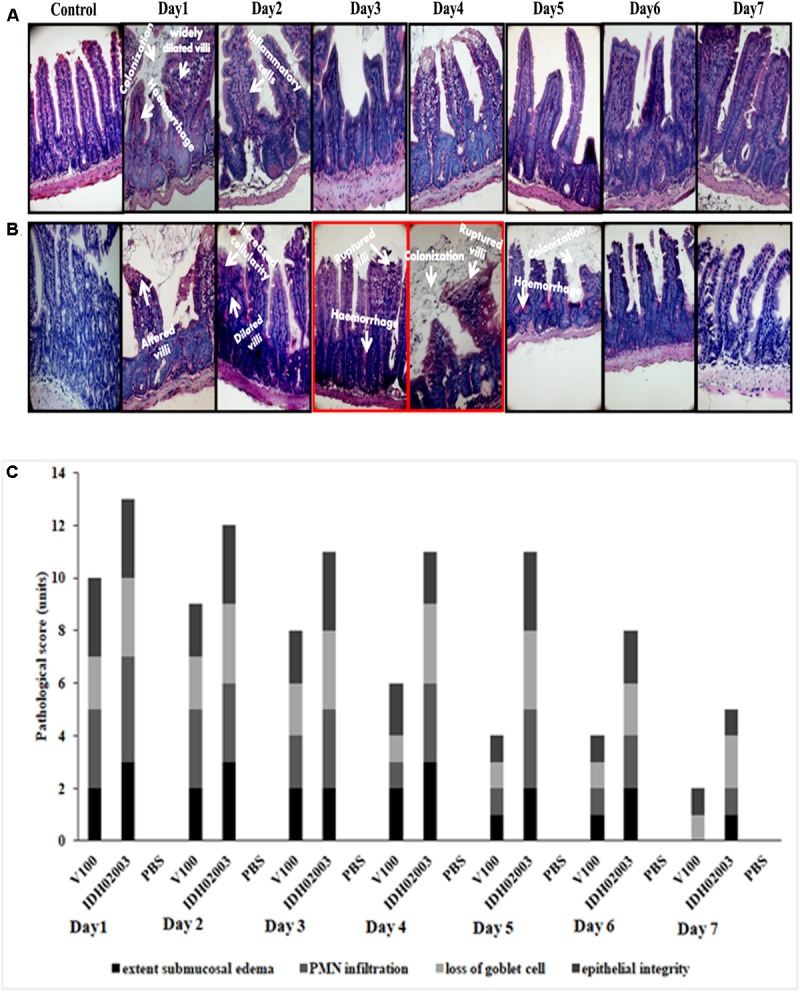
Hematoxylin and Eosin – stained sections of mice intestine challenged with *V. cholerae* strains. **(A)** Log phase *V. cholerae* El Tor strain V100 (1 × 10^9^) was inoculated and mice intestine were collected for H&E staining as described in Section “Materials and Methods” over a 7-day period. **(B)** Log phase *V. cholerae* Haitian variant strain IDH02003 (1 × 10^9^) was inoculated and mice intestine were collected for H&E staining as described in Section “Materials and Methods” for 7-day period. In each case uninfected mice were used as control. As can be seen, mice infected with V100 showed widely dilated villi with congestion, hemorrhage, and increased cellularity with bacterial colonization on day 1. Inflammatory changes were present to some extent up to day 2 which gradually become normal day 3 onwards. On the other hand IDH02003 showed higher virulence with increased mucosal damaging, inflammation, and colonization ability till days 4–5. Gross structural alteration with distorted villous epithelium was observed even on day 5. From day 6, severity of lesion gradually declined leading to almost normal on day 7. **(C)** H&E stained sections scored on the basis of Congestion and Inflammation.

**FIGURE 9:**
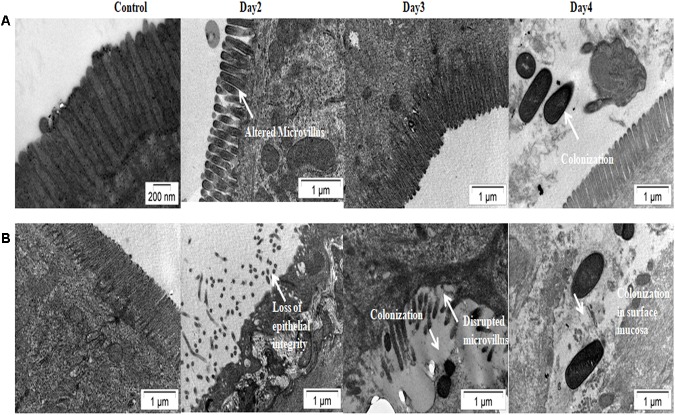
Transmission electron microscopy of mice intestine challenged with *V. cholerae* strains. **(A)** Log phase *V. cholerae* El Tor strain V100 (1 × 10^9^) was inoculated and mice intestine were collected for TEM analysis as described in Section “Materials and Methods.” **(B)** Log phase *V. cholerae* Haitian variant strain IDH02003 (1 × 10^9^) was inoculated and mice intestine were collected for TEM analysis as described in Section “Materials and Methods.” In each case uninfected mice were used as control. Infection with V100 showed altered microvillus structure on day 2, which comes to almost normal stage on day 3. Colonization of bacteria was seen on day 4. As can be seen that infection with IDH02003 on the other hand showed gross disruption of the microvillus structure on day 2. On day 3 along with disruption, bacterial colonization was observed.

Results of bacterial shedding and colonization motivated us to analyze the intestinal tissue damaging ability of the Haitian variant strains. Histopathological staining of the mouse tissue infected with V100 showed widely dilated villi with congestion, hemorrhage and increased cellularity with bacterial colonization on day 1. Inflammatory changes were observed to some extent up to day 2 and turned to normal from day 3 onward. On the other hand, IDH02003 showed increased mucosal damaging, inflammation and colonization ability until days 4–5. Gross structural alteration with distorted villus epithelium could be seen even on day 5. Day 6 onward, severity of the lesion gradually declined and became almost normal on day 7 (Figure 8A–C). TEM analysis with the same intestinal tissues has shown the greater damaging potential of IDH02003 and IDH00990 with higher colonization and the ability to disrupt the microvillus structure compared to V100 and V114 (Figure 9A,B).

## Discussion

The Haitian cholera epidemic was a dreadful event among recent cholera epidemics. The rapidity and severity of this epidemic pointed to the need for a close monitoring of the emergence and the dissemination of Haitian variant strains. Comparative studies demonstrated the unique genomic composition of the Haitian variant strain that might have helped the pathogen to gain greater ecological fitness and infectivity (Grim et al., 2010). Our earlier studies showed that Haitian variant strain possessed differences in the virulence conferring genes, including *ctxB*, *tcpA*, and *rtxA* (Naha et al., 2012; Ghosh et al., 2014a,b). This variant has been a major cause of recent outbreaks. They were predominant in most of the cholera endemic regions of India and Bangladesh (Ghosh et al., 2014b; Kumar et al., 2014; Rashid et al., 2016). These strains have spread across all major continents but the clinical relevance of these genetically variant strains is limited. Increased lethality was reported in infant mouse model for single strain infection study with Haitian variant compared to El Tor (Son et al., 2011). Increased colonization fitness in infant mice for Haitian variant was published (Satchell et al., 2016). Higher fluid accumulation in rabbit ileal loop infected with El Tor variants were also documented (Ghosh-Banerjee et al., 2010). Canonical El Tor and Haitian variant strains were tested in this study to understand the colonization ability and virulence in different animal models.

Colonization ability marks the adherent capacity of *V. cholerae* on intestinal epithelium, which is essential for its pathogenesis (Almagro-Moreno et al., 2015). Significantly higher colonization was observed with Haitian variant strains when compared with the canonical El Tor strains in the infant mouse model. Thus, it appeared that the Haitian variant strain with its altered genomic constitution can adhere more efficiently, which in turn can lead to higher level of CT secretion. To confirm this observation, fluid accumulation in rabbit intestine was examined. The Haitian variant strain was found to cause significantly higher hemorrhagic fluid accumulation compared to the canonical El Tor strain. Colonization ability was also analyzed in the same rabbit model and was found to be similarly significantly higher in the Haitian variants. The results of these virulence assays were further tested for diarrheal persistency in adult mouse model. Persistent diarrhea was observed with Haitian variant, which was totally different from the gradually decreasing pattern observed with the El Tor strains. Interestingly, the shedding pattern of the Haitian variant strain resembled to that of non-O1 and non-O139 *V. cholerae* strains (Dziejman et al., 2005). Adult mouse colonization results again validated persistent diarrhea caused by Haitian variant. Further, histopathology and TEM results confirmed the hyper-virulence expression by Haitian variant strains. All these observations showed that the Haitian variant strains are more pathogenic. It is also believed that virulence factors, colonization ability and CT could influence the symbiotic and/or commensal association between *V. cholerae* and specific aquatic organisms (Faruque et al., 2003), which in turn may help the organism to persist in the environment more efficiently.

With the appearance of this Haitian variant, there has been a subtle but distinct change in the cholera epidemiology in recent years. These differences include an increase in the severity of the disease as compared to those caused by canonical El Tor, the tendency of the epidemics to linger longer as has been seen recently in Zimbabwe, Haiti, and Yemen (Islam et al., 2011; Satchell et al., 2016; Rabaan, 2018). There is a constant need for identifying and tracking these strains to implement appropriate measures and to contain the outbreaks and epidemics and further studies should be focused for better understanding the potential of these hyper-virulent strains in human-to-human spread, the severity of disease, and the environmental persistence.

## Author Contributions

PG, AM, and SD conceived and designed the experiments. PG, RS, PS, and HK performed the experiments. PG, AM, DS, and KO analyzed the data. AM, TR, SD, and KO contributed reagents, materials, and analysis tools. PG, AG, TR, and AM wrote the manuscript. All authors read and approved the final manuscript.

## Conflict of Interest Statement

The authors declare that the research was conducted in the absence of any commercial or financial relationships that could be construed as a potential conflict of interest.
